# Adherence to Guidelines in Orthopaedic Operation Notes: A Quality Improvement Initiative

**DOI:** 10.7759/cureus.84632

**Published:** 2025-05-22

**Authors:** Mohd A Aslam, Devashish Chhutani, Vineet Kumar, Swagat Mahapatra, Pankaj Aggarwal

**Affiliations:** 1 Department of Orthopaedic Surgery, Dr. Ram Manohar Lohia Institute of Medical Sciences, Lucknow, IND

**Keywords:** clinical audit, dvt, operative notes, rcs guidelines, standardized templates

## Abstract

Background: Orthopaedic operation notes are crucial documents that record the specifics of surgical procedures performed on the musculoskeletal system. They play a vital role in ensuring clear communication between peri-operative and post-operative periods, maintaining patient care and safety, and serving as legal documents. However, studies have consistently shown gaps in the documentation of important parameters.

Objective: This study aimed to compare current practices in documenting orthopaedic operation notes with changes in these practices after awareness training and the implementation of an operative notes template, as per the Royal College of Surgeons of England's (RCS) guidelines.

Methods: A closed-loop, two-cycle clinical audit was conducted at Dr. Ram Manohar Lohia Institute of Medical Sciences Hospital, Lucknow, India. The first cycle retrospectively analyzed 100 randomly selected surgical notes from December 2024, while the second cycle prospectively analyzed 100 randomly selected surgical notes from March 2025, following a two-month training program and implementation of a new proforma.

Results: The audit showed initial high compliance rates for patient identification (96%) and consultant-in-charge and anaesthetist details (90%), but low rates for all other key parameters, the compliance for a few of which, like surgical details, complications, DVT prophylaxis, and post-operative instructions, was alarmingly low. After targeted efforts, significant improvements were seen, notably in documentation of surgery date/time (91%), emergency/elective procedures (86%), and operative findings (76%). Compliance rates for complications, extra procedures, and post-op care also rose, ranging from 60% to 90%. Overall, documented parameters increased from 35.8% to 90.2%. A statistical analysis comprising a paired-sample *t*-test and a Z-test for comparing confirmed that the improvement was significant.

Conclusion: The introduction of proper training and implementation of a new proforma based on RCS guidelines led to remarkable improvements in the quality of operative notes. This study highlights the need for regular audits, implementation of standardized templates, and consideration of electronic documentation systems to improve documentation practices.

## Introduction

In the realm of healthcare, meticulous documentation of surgical procedures is crucial for ensuring patient safety and effective communication among medical professionals. Orthopaedic operation notes, serving as the official record of surgical interventions, directly impact patient safety, continuity of care, and legal accountability. These notes also serve as a vital communication tool among healthcare providers, ensuring accurate relay of critical information [1,2].

Operation notes must adhere to standardized guidelines, including essential elements such as patient identification, preoperative diagnosis, operative procedure, surgical findings, and postoperative plans. Incomplete or inaccurate documentation can lead to adverse events, misunderstandings, and potential legal issues. Essential details like prophylactic measures, complications, deep vein thrombosis (DVT) prophylaxis, and antibiotic dosage and prophylaxis must be accurately documented [2].

Despite guidelines from authoritative bodies, alarming lapses in documentation persist, compromising postoperative care, introducing medical errors, and undermining institutional accountability.

Also, freehand notes without a template pose significant risks to patient safety and care. They can be difficult to interpret, potentially leading to errors, and incorrect surgical item counts may result in retained foreign objects [2-4]. Non-standardized abbreviations can cause confusion, while the quality of notes relies heavily on the author's detail, legibility, and adequacy. Poor documentation in healthcare can lead to medical errors, compromised patient safety, and medico-legal issues, including malpractice lawsuits and financial losses. In medico-legal cases, the inadequacy of freehand notes can be particularly problematic, with up to 45% of operative notes deemed indefensible in court [5].

The gaps identified in this audit, such as incomplete documentation of complications, surgical details, and postoperative care, can directly compromise postoperative care, increase the risk of medical errors, and undermine institutional accountability. The implementation of standardized documentation practices, potentially through structured templates or guidelines, is essential for protecting patient safety and ensuring consistent quality care.

Auditing clinical practice is crucial for maintaining and enhancing the quality of healthcare services. In orthopaedic surgery, precise and consistent documentation in operation notes is vital for guiding postoperative care, facilitating communication among healthcare providers, and providing a medico-legal record of surgical procedures [5]. Previous studies have highlighted significant issues in surgical documentation globally, emphasizing the need for regular audits and adherence to standardized protocols [3,4,5].

This review aims to evaluate current practices in orthopaedic operation note documentation, identify gaps in compliance with established guidelines, and explore potential interventions to improve adherence to standards. By examining existing literature and best practices, we can inform strategies to enhance the quality and safety of orthopaedic surgical care.

## Materials and methods

The audit was conducted at Dr. Ram Manohar Lohia Institute of Medical Sciences Hospital, Lucknow, India, a 1,200-bed interdisciplinary tertiary care institution, to evaluate the compliance of orthopaedic surgical documentation with the Royal College of Surgeons (RCS) Good Surgical Practice guidelines. Institutional approval was secured from the Institutional Clinical Audit Committee (ERC), ensuring adherence to ethical standards congruent with the Declaration of Helsinki for research involving human subjects [6].

The study employed a closed-loop audit methodology, consisting of both retrospective and prospective data collection phases. The study was conducted over a duration of four and a half months, divided into five phases: (1) conceptualization and research note submission (December 15-31, 2024); (2) retrospective data collection (December 2024, 31 days); (3) intervention phase, including training and introduction of a new proforma (January to February 2025, 2 months); (4) prospective data collection (March 2025, 1 month); and (5) data analysis and final report preparation (15 days).

In the initial phase of the retrospective data collection, spanning one month from December 1, 2024, to December 31, 2024, a total of 100 handwritten operation notes, comprising 50 patients from elective and emergency procedures each, were randomly selected using a simple randomization method. These notes were assessed retrospectively using computer-generated random allocation, ensuring an unbiased representation of the data. A sample size of 100 operation notes was calculated to determine the appropriate number of observations needed to provide sufficient statistical power and precision for the study's objectives and to establish differences among variables. The study included all operated patients without any exclusion criteria, ensuring a comprehensive representation of the population. This inclusive approach allowed for a broader understanding of the documentation practices across various cases.

Each note was reviewed for specific elements outlined by the Good Surgical Practice guidelines from the RCS [2,3] and an article from the *British Medical Journal* [3]. The Good Surgical Practice guidelines outline essential elements for surgical documentation, identified through a comprehensive review of existing literature and guidelines from authoritative bodies. The elements included the identities of the surgical team, such as the consultant in charge, operating surgeon, assisting surgeon, and anesthetist, diagnostic information, surgical procedure specifics, and postoperative care directives. The notes were rigorously compared against the RCS guidelines to assess completeness, accuracy, and quality, with all reviews performed by a single reviewer.

Particular attention was given to verifying the inclusion of crucial elements such as the date and time of the operation, identification of the medical personnel involved, the nature of the procedure (whether elective or emergency), operative diagnoses, incision details, application of tourniquets, any intraoperative complications, and implant details, including the company material and serial numbers. The affixation of signatures was also assessed (Table 1).

**Table 1:** Parameters of complete operative note as suggested by the Royal College of Surgeons (RCS) guidelines [2,3].

Identifying parameters	Surgical parameters	Post-operative parameters
Hospital number	Patient position	Antibiotic prophylaxis
Patient identifiers	Exposure	Deep vein thrombosis prophylaxis
Date/time	Incision	Weight-bearing status
Handwritten vs typed	Operative diagnosis	Follow up
Legible (grades 1-4)	Operative findings	Post-operative instructions
Consultant name	Extra procedures with reasoning	Prosthesis labels
Surgeon name	Tissue removed/added/altered	Signature
Grade of operating surgeon	Intraoperative complications	
Assistant name	Tourniquet time	
Anaesthetist name	Details of closure	
Type of anaesthesia	Anticipated blood loss	
Emergency/scheduled		
Procedure name		
Side		
Indication		
Procedure		

**Figure 1:**
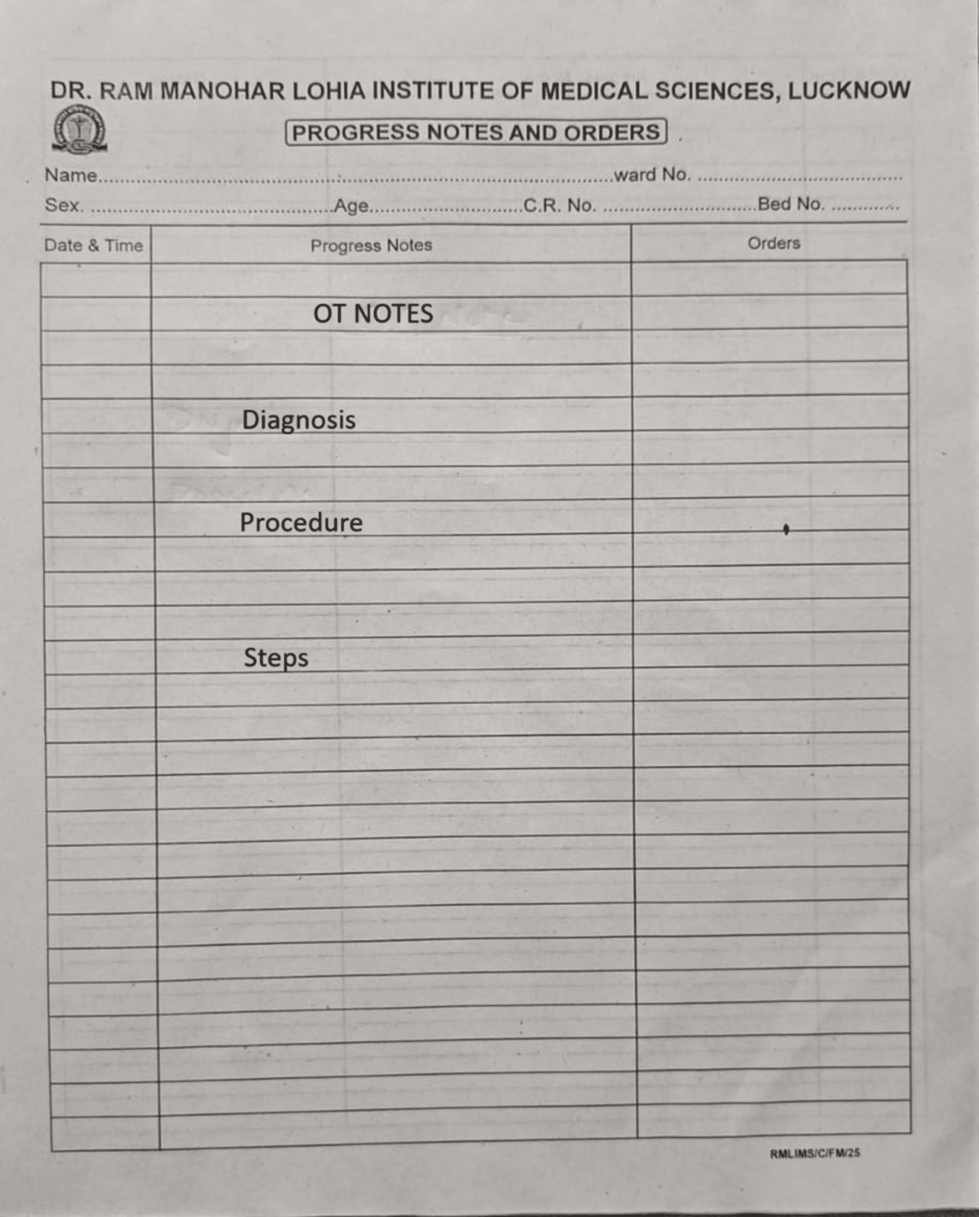
The initial operation notes proforma

**Figure 2:**
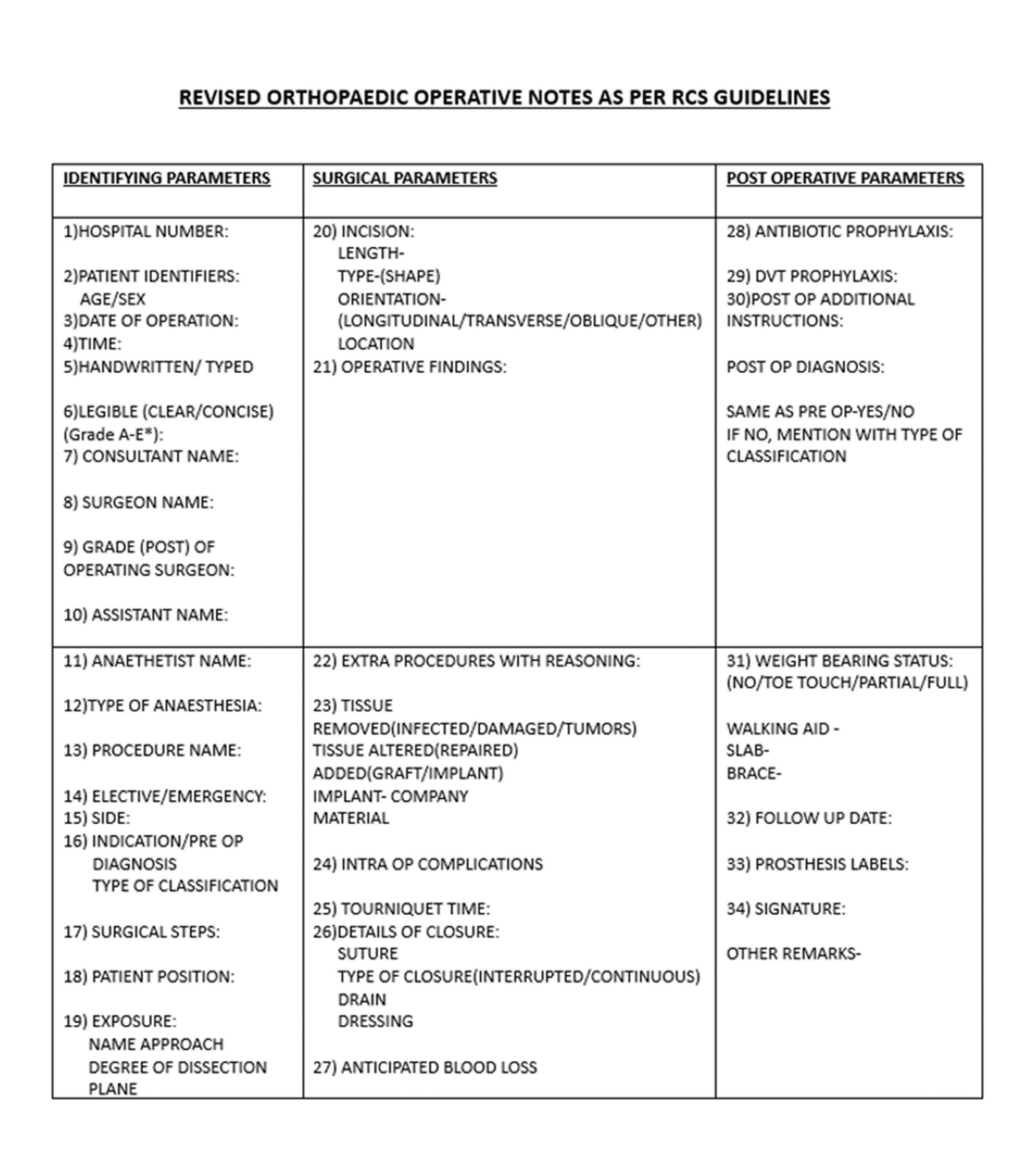
The new operation notes proforma picture DVT: deep vein thrombosis.

Data were entered into Excel spreadsheets (Microsoft Corporation, Redmond, Washington) and analyzed using paired-sample t-tests to compare means and Z-tests to compare proportions. Statistical significance was determined by a p-value of less than 0.05, indicating meaningful differences between groups. The findings, including missing details and incompleteness, led to the implementation of training in the form of questionnaires and training classes during the third phase, lasting for two months in January and February 2025. The training program included a comprehensive questionnaire covering demographics, documentation practices, challenges, and training needs, aiming to improve documentation practices (Table 2). This was followed by discontinuation of the old version of the operative note, known as the progress sheet (Figure 1), and the introduction of a new proforma (Figure 2). An awareness session targeted individuals such as residents and consultant surgeons who were actively involved in the documentation process. Use of the new proforma became mandatory, replacing the old version.

**Table 2:** Training and discussion topics based on Royal College of Surgeons of England (RCS) guidelines

Topics
Section 1: Demographics
Section 2: Current documentation practices – Currently use a standardized template for documenting operative notes, information to be included in your operative notes, when, and what to document
Section 3: Knowledge of RCS guidelines – Familiarity, training, awareness, and ability to document as per RCS guidelines
Section 4: Challenges in documentation – Time constraints, lack of standardized template, difficulty in documenting complex cases, Illegible handwriting, limited access to electronic documentation systems
Section 5: Training needs – Additional training on documenting operative notes, proforma, regular updates, key areas, gaps in documentation
Section 6: Further suggestions and open discussion

In the fourth phase of prospective data collection, covering around one month from March 1, 2025, to March 31, 2025, the same number of patients were selected randomly from the surgical records, comprising both elective and emergency ward patients, with 50 patients in each group as before, without any exclusion criterion. The operation notes were assessed, and the parameters from the second dataset were analyzed alongside the pre-intervention measures. The results were presented to the local audit committee, which expressed satisfaction with the improvements. Following this, the changes were implemented as standard practice, completing the audit.

Statistical analysis

For the analysis of the data, the latest version of the IBM SPSS Statistics for Windows, Version 25 (Released 2017; IBM Corp., Armonk, New York) was utilized. Statistical analysis involved a paired-sample t-test to compare means and a Z-test for comparing proportions. A p-value of less than 0.05 was considered statistically significant, indicating meaningful differences between groups.

## Results

A two-cycle audit was conducted at Dr. Ram Manohar Lohia Institute of Medical Sciences Hospital to evaluate orthopaedic surgical documentation compliance with RCS guidelines. The initial audit showed high compliance rates for patient identification (96%) and consultant/anesthetist details (90%). However, parameters such as emergency or elective procedures, note legibility, complications, and extra procedure documentation had zero compliance, most likely due to the absence of specific fields or reminders in the previously used proforma. This highlighted the need for a more comprehensive documentation template. The names of the operating surgeon and assistant were documented 50% and 64% of the time, respectively. Operative findings, preoperative diagnosis, and patient position had low compliance rates, ranging from 50% to 60%. Surgical parameters such as surgical planes, blood loss, tourniquet time, and closure details had compliance rates below 30%. The implant company was mentioned in 50% of the cases, but the implant material was only noted in 10%. The date and time of surgery were missing in 30% of cases (Table 3).

**Table 3:** Comparison of RCS guideline parameters compliance percentage before and after training RCS: Royal College of Surgeons of England, DVT: Deep vein thrombosis.

Parameter assessed	Compliance percentage		Percentage of improvement in compliance	P-value
	Pre-training documentation frequency	Post-training documentation frequency		
Identification age/sex	96	99	3	0.322
Date and time of surgery	60	91	31	0.021
Legibility of notes	0	81	81	<0.001
Consultant in charge's name	90	98	8	0.081
Operating surgeon name	50	88	38	0.002
Grade(post)of operating surgeon	40	84	44	<0.001
Assistant name	64	88	24	0.002
Anaesthetist name	90	96	6	0.061
Type of anaesthesia	54	88	34	0.02
Emergency/elective	0	86	86	<0.001
Procedure name	70	92	22	0.027
Side	68	94	26	0.021
Operative diagnosis	66	90	24	0.021
Operative findings	52	76	24	0.023
Position of the patient	56	92	36	0.002
Incision shape	24	68	44	<0.001
Surgical planes	22	76	54	<0.001
Complications	0	60	60	<0.001
Extra procedure documentation with reasoning	0	80	80	<0.001
Details of tissues added/altered/removed	42	80	38	<0.001
Closure technique details	5	76	71	<0.001
Anticipated blood loss	24	82	58	<0.001
Tourniquet time	20	78	58	<0.001
Implant material and other details	10	82	72	0.001
Antibiotic prophylaxis	68	90	22	0.041
DVT prophylaxis (where applicable)	21	84	63	<0.001
Post op instructions	70	90	20	0.023
Weight-bearing protocol	10	74	64	<0.001
Follow-up	50	78	28	0.014
Prosthesis labels/company	50	88	38	<0.001
Signature	70	98	28	0.013

Following targeted efforts to enhance documentation, significant improvements were observed in various parameters. Notably, compliance rates improved for key aspects such as the date and time of surgery, which reached 91%, and documentation of elective or emergency procedures, which rose to 86%. The accuracy of operative findings also saw a considerable increase, reaching a compliance rate of 76%. Furthermore, substantial improvements were noted in the documentation of complications, extra procedures, and surgical notes, with compliance rates ranging from 60% to 90%. Additionally, postoperative parameters, including instructions, weight-bearing status, and details regarding implants, demonstrated compliance rates exceeding 70%.

**Figure 3:**
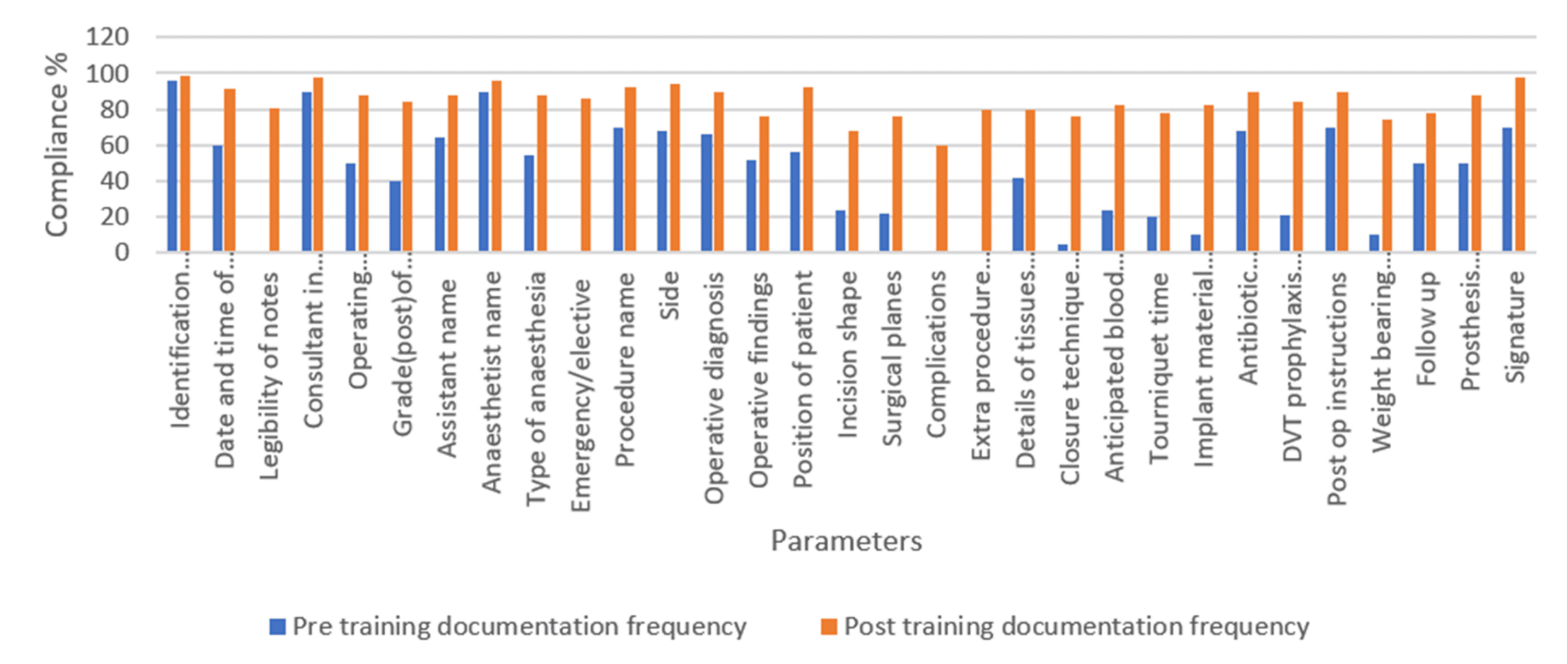
Comparison of pre- and post-training documentation compliance frequency expressed in percentage The bar diagram shows improvement in compliance of all parameters. DVT: deep vein thrombosis.

The audit demonstrated a significant increase in documentation quality, with 90.2% of parameters now documented compared to 35.8% initially, reflecting a marked improvement in communication and safety standards in orthopaedic care. Paired-sample t-test results confirmed a significant and large difference in documentation quality before and after introducing the new proforma. These findings highlight the effectiveness of targeted efforts in improving surgical record-keeping and compliance with guidelines (Figure 3).

## Discussion

With the increasing number of surgical procedures being performed worldwide, it is crucial for surgeons to adhere to the guidelines set by the RCS for creating high-quality operative notes [1,2]. These guidelines play a vital role in ensuring that patient care is optimized after surgery and provide a layer of legal protection for surgeons [5]. However, past research [7-11] has consistently shown that many surgical specialties struggle to fully implement these guidelines, resulting in incomplete or inadequate documentation. Our audit at this institution examined how well orthopaedic surgical notes complied with RCS standards and revealed significant shortcomings. Specifically, the generic progress sheets used for operation notes failed to capture the unique details required for orthopaedic procedures, leading to critical omissions.

The audit revealed significant gaps in documentation, with only 35.8% of parameters documented and a mean compliance rate of 38%. This finding is consistent with studies by Ngibo et al. [7], which reported a low compliance rate of 47.4%. The suboptimal compliance may be attributed to factors such as a lack of formal training in writing operation notes, inadequate design of operation notes, and insufficient staffing, leading to increased stress on available surgeons.

Several parameters had low compliance rates, including postoperative instructions, weight-bearing status, need for extra stabilization support, and implant details. These findings are supported by studies conducted by Khan et al. [8] and Pandor et al. [9]. However, a retrospective study by Paul et al. [10] reported better compliance rates in documentation of parameters, except for DVT prophylaxis. A possible explanation for this discrepancy might be the relatively lighter workload and fewer admissions and operations performed during regular hours in their study (n = 75 over 5 months).

The inadequate design of the operation notes likely contributed to the 0% documentation rate for certain parameters, such as elective or emergency procedures, note legibility, complications, and extra procedure documentation.

Our study implemented a comprehensive intervention, including a two-month training program with classes and local presentations to educate staff on standard operation note contents, a questionnaire to assess knowledge improvements, and the design of a new operation note proforma incorporating Royal College of Surgeons guidelines. An RCS checklist was also placed in each file to ensure compliance. Following the intervention, significant improvements were observed, with the overall compliance rate rising from 35.8% to 90.2%. Documentation of elective or emergency procedures improved from 0% to 86%, and substantial gains were seen in anticipated blood loss, antibiotic prophylaxis, and DVT prophylaxis documentation. These findings are consistent with similar audits by Khan et al. [8] and Hassan et al. [11], which demonstrated notable enhancements in compliance after redesigning operation note templates.

Targeted interventions significantly improved surgical documentation, with notable increases in operative findings, complication reporting, and consistent documentation of surgical team members, procedure details, and postoperative care instructions. Document validation via signatures also approached universal compliance. To further enhance the system, implementing a computer-generated template with typed operation notes would address omissions and illegibility issues [12]. A quality improvement project is underway to develop software with dropdown menus for typed operative notes, accessible to all surgical specialties, aiming for a more permanent solution [13].

Limitations

This study has several limitations, including the lack of a second reviewer to cross-check notes, which may have introduced reviewer bias and impacted data consistency and reliability. Additionally, the single-centric design and small sample size may limit generalizability. Future studies with larger sample sizes and multicenter involvement are warranted to validate the findings and enhance the reliability of results, ideally incorporating inter-rater reliability checks to ensure data accuracy.

## Conclusions

The audit revealed significant gaps in orthopaedic operation note documentation, compromising patient safety and care continuity. To address this, adopting standardized templates, leveraging electronic health records for real-time data entry, and providing targeted training programs for surgical teams are recommended. Regular audits can help identify areas for improvement, while integrating typed operative note systems into hospital software can enhance record-keeping accuracy and efficiency. By implementing these measures, healthcare institutions can improve documentation practices, enhance patient safety, ensure regulatory compliance, and promote quality improvement in surgical disciplines, ultimately leading to better postoperative outcomes and reduced complications.
